# A First-Order Differential Data Processing Method for Accuracy Improvement of Complementary Filtering in Micro-UAV Attitude Estimation

**DOI:** 10.3390/s19061340

**Published:** 2019-03-18

**Authors:** Xudong Wen, Chunwu Liu, Zhiping Huang, Shaojing Su, Xiaojun Guo, Zhen Zuo, Hao Qu

**Affiliations:** College of Intelligence Science and Technology, National University of Defense Technology, Changsha 410073, China; wenxudong13@163.com (X.W.); kdhuangzp@163.com (Z.H.); susj-5@163.com (S.S.); jeanakin@nudt.edu.cn (X.G.); z.zuo@nudt.edu.cn (Z.Z.); quhao199541@163.com (H.Q.)

**Keywords:** attitude estimation, nonlinear complementary filtering (NCF), sensor fusion, micro-UAV, data processing

## Abstract

There are many algorithms that can be used to fuse sensor data. The complementary filtering algorithm has low computational complexity and good real-time performance characteristics. It is very suitable for attitude estimation of small unmanned aerial vehicles (micro-UAVs) equipped with low-cost inertial measurement units (IMUs). However, its low attitude estimation accuracy severely limits its applications. Though, many methods have been proposed by researchers to improve attitude estimation accuracy of complementary filtering algorithms, there are few studies that aim to improve it from the data processing aspect. In this paper, a real-time first-order differential data processing algorithm is proposed for gyroscope data, and an adaptive adjustment strategy is designed for the parameters in the algorithm. Besides, the differential-nonlinear complementary filtering (D-NCF) algorithm is proposed by combine the first-order differential data processing algorithm with the basic nonlinear complementary filtering (NCF) algorithm. The experimental results show that the first-order differential data processing algorithm can effectively correct the gyroscope data, and the Root Mean Square Error (RMSE) of attitude estimation of the D-NCF algorithm is smaller than when the NCF algorithm is used. The RMSE of the roll angle decreases from 1.1653 to 0.5093, that of the pitch angle decreases from 2.9638 to 1.5542, and that of the yaw angle decreases from 0.9398 to 0.6827. In general, the attitude estimation accuracy of D-NCF algorithm is higher than that of the NCF algorithm.

## 1. Introduction

Micro-UAVs have the advantages of low cost, good concealment and strong survivability. They have broad application prospects in military and civil fields such as scientific research, ecological protection and economic construction. Accurate attitude estimation is the basis for flight control of micro-UAVs. In recent years, the development of microelectromechanical system (MEMS) technology has further reduced the size and cost of inertial measurement units (IMUs), making MEMS IMUs widely used in micro-UAVs [1]. Compared with normal sensors, the performance of MEMS sensors is poor. Therefore, the attitude accuracy estimated by MEMS IMUs is low. How to improve the accuracy of attitude estimation of MEMS IMUs has become a hot topic in recent years [2].

IMUs usually consist of a three-axis gyroscope, a three-axis accelerometer and a three-axis magnetometer [3]. Gyroscope measurements contain time drift and noise, it is not accurate to directly integrate of the angular rate measured by gyroscope. However, its dynamic performance is good; Accelerometer measurements contain high-frequency noise and it is sensitive to motion acceleration. Thus, when a micro-UAV is hovering or accelerating, large errors will be produced in measurements. The advantage of accelerometer is that its static performance is good which means its output does not drift with time; Magnetometers are susceptible to electronic noise from electronic or ferromagnetic equipment [4]. In order to overcome the shortcomings of each sensor and improve the accuracy of UAV’s attitude estimation, researchers have proposed many algorithms to achieve data fusion of IMU sensors, which mainly can be divided into three categories [5]: (1) stochastic approach (Kalman filtering); (2) deterministic approach (Wahba’s problem); and (3) frequency-based approach (Complementary filtering).

The attitude estimation technique based on Kalman filter has been developed for a long time. To compensate the shortcoming of Kalman filtering that it cannot solve non-linear problems, many improved algorithms have also been applied to UAV attitude estimation, such as the Extended Kalman Filter (EKF), Unscented Kalman Filter (UKF), and Cubature Kalman Filter (CKF) [6,7], etc. The accuracy of these algorithms is high, but there are linearization errors, truncation errors and divergence problems [8]. Moreover, these algorithms strongly depend on noise estimation. When the noise model or noise covariance is not accurate, the attitude estimation performance will be greatly affected [9]. Deterministic approach tries to obtain a best fit attitude estimation from sensor measurement, such as Gradient Descent Algorithm (GDA) [10], Gauss-Newton Algorithm (GNA) [11], etc. These algorithms require multiple iterations and computationally time consuming matrix inverse operations [12]. They aren’t suitable for micro-UAVs whose computing resources is limited. Compared with the previous two methods, the frequency-based method (complementary filtering algorithm) is computationally simple [13]. It is very suitable for micro-UAVs with limited computing resources. However, the accuracy of the attitude estimation using complementary filtering algorithms is poor, which is the biggest obstacle to their wide application in micro-UAVs.

In order to improve the accuracy of the attitude estimation of complementary filtering algorithms, scholars have proposed many improved methods. In [14], a multiple model adaptive complementary filter algorithm is proposed. In [15], Poddar et al. studied the combination of PSO algorithm and complementary filtering algorithm. In [16], Li et al. designed a method for correcting external acceleration. In [17], EKF and complementary filter are combined to solve the attitude estimation problem. The above methods improve the accuracy of the complementary filtering algorithm to a certain extent, but the calculation complexity is greatly increased. Besides, these algorithms work normally on the premise that sensor measurements are reliable. Obviously, if there is an accidental error in the sensor data, the effect of these algorithm will be greatly affected. 

Some scholars have also studied the data processing methods in attitude estimation. The proposed data preprocessing methods for gyroscopes include Allan variance analysis, time-series analysis, Kalman filtering [18], etc. The core of these algorithms is to analyze a lot of gyroscope data in advance, and then design a suitable filter for real-time filtering. It’s obvious that these algorithms have poor portability and flexibility, and the workload is large. Besides, some scholars have done some research about the data robustness in UAV attitude estimation. For example, in [19], the author proposed to use the Set-Based Approach to achieve fault detection and isolation (FDI) based on the dual IMU platform. In [20], a combination of a single Kalman filter and a Gaussian hidden Markov model for each of the monitored sensors allows to simultaneously detect single and multiple sensor faults. However, the versatility of these fault detection algorithms is not good and their calculations are complex. 

In general, there are relatively few studies on improving the accuracy of complementary filtering algorithms from the data processing aspects. What’s more, there is a lack of a real-time and robust data processing algorithm for gyroscopes. To further improve the performance of complementary filtering algorithm in micro-UAVs attitude estimation. In this paper, a real-time first-order differential data processing algorithm is proposed for gyroscope data, and an adaptive adjustment strategy is designed for the parameters in the algorithm. Besides, the D-NCF algorithm is proposed by combine the data processing algorithm with the basic NCF algorithm. The experimental results show that the first-order differential data processing algorithm can effectively correct the gyroscope data, which guarantee the robustness, and the D-NCF algorithm greatly improve the accuracy of the complementary filtering algorithm. In general, the proposed algorithm has practical value in engineering.

## 2. Attitude Estimation Based on Complementary Filtering

### 2.1. Attitude Description

The attitude of the UAV is described by three attitude angles: pitch, yaw, and roll, which are represented by γ, *θ* and ψ. The navigation coordinate system (N) is defined as the East-North-Up (ENU) coordinate system, and the body coordinate system (B) is defined as the Right-Forward-Up (RFU) coordinate system. The essence of attitude estimation is to calculate the relative position of the body coordinate system (B) to the navigation coordinate system (N).

The attitude description methods mainly include Euler angle, direction cosine and quaternion [21]. Euler angle is simple and easy to express, but there are singularities. Direction cosine can meet the requirements of omnidirectional attitude estimation, but the calculation amount is relatively large. In contrast, the quaternion description can well overcome the above problems, the amount of calculation is moderate and there is no singularity, it is a very efficient attitude description method.

Assume that the quaternion vector $q_{I}={\left[q_{0},q_{1},q_{2},q_{3}\right]}^{T}$ represents the direction of the B system relative to the N system. Then any vector *x* in the N system can be converted into the B system by the following formula:

(1) $$x^{B}=C_{N}^{B}(q_{I})x^{N}$$

where, the rotation matrix $C_{N}^{B}(q_{I})$ between the N system and the B system is defined as:

(2) $$C_{N}^{B}(q_{I})=\left[\begin{array}{ccc} q_{0}^{2}+q_{1}^{2}-q_{2}^{2}-q_{3}^{2} & 2(q_{1}q_{2}+q_{0}q_{3}) & 2(q_{1}q_{3}-q_{0}q_{2})\\ 2(q_{1}q_{2}-q_{0}q_{3}) & q_{0}^{2}-q_{1}^{2}+q_{2}^{2}-q_{3}^{2} & 2(q_{2}q_{3}+q_{0}q_{1})\\ 2(q_{1}q_{3}+q_{0}q_{2}) & 2(q_{2}q_{3}+q_{0}q_{1}) & q_{0}^{2}-q_{1}^{2}-q_{2}^{2}+q_{3}^{2} \end{array}\right]$$

Usually, after the quaternion is updated in real time by the attitude estimation algorithm, it needs to be converted into Euler angles for the convenience of controller processing. The formula for the transformation from quaternion to Euler angle is:

(3) $$\{\begin{array}{l} \gamma =\arctan\frac{2(q_{2}q_{3}+q_{0}q_{1})}{q_{0}^{2}-q_{1}^{2}-q_{2}^{2}+q_{3}^{2}}\\ \theta =-\arcsin2(q_{1}q_{3}-q_{0}q_{2})\\ \psi =\arctan\frac{2(q_{1}q_{2}+q_{0}q_{3})}{q_{0}^{2}+q_{1}^{2}-q_{2}^{2}-q_{3}^{2}} \end{array}$$

### 2.2. Attitude Estimation by Gyroscope

The output data of the gyroscope contains noise. Even if the noise is very weak, after long time integration, a large attitude error will occur. The gyroscope output model can be expressed as [22]:

(4) $$\omega_{g}=\omega +b+\eta$$

where, ω is the actual angular velocity, b is the three-axis drift of the gyroscope, and η is the Gaussian white noise whose mean is zero and influence on the attitude is negligible after integration. Therefore, η=0 is assumed in the following derivation, and then the above formula becomes:

(5) $$\omega_{g}=\omega +b$$

Under the assumption of noiselessness, attitude estimation result can be obtained by gyroscopes alone. The method is to update the quaternion by solving the following dynamic equation according to the angular velocity $\omega_{g}=\left[\omega_{gx},\omega_{gy},\omega_{gz}\right]$ measured by gyroscopes [23]:

(6) $${\dot{q}}_{I}=\frac{1}{2}Q\left(\omega \right)⊗\omega_{B}$$

Substituting $q_{I}$ and $\omega_{g}$ into the above formula:

(7) $$\left[\begin{array}{c} {\dot{q}}_{0}\\ {\dot{q}}_{1}\\ {\dot{q}}_{2}\\ {\dot{q}}_{3} \end{array}\right]=\frac{1}{2}\left[\begin{array}{cccc} q_{0} & -q_{1} & -q_{2} & -q_{3}\\ q_{1} & q_{0} & -q_{3} & q_{2}\\ q_{2} & q_{3} & q_{0} & -q_{1}\\ q_{3} & -q_{2} & q_{1} & q_{0} \end{array}\right]\left[\begin{array}{c} 0\\ \omega_{gx}\\ \omega_{gy}\\ \omega_{gz} \end{array}\right]$$

In general, the above differential equation can be solved by a (single-sample, multi-sample) rotation vector method, a Picard approximation method, or a (first-order, second-order, fourth-order) Runge-Kutta algorithm [24]. After obtaining the updated quaternion, the Euler angle can be further calculated based on Equation (3).

### 2.3. Attitude Estimation by Accelerometer/Magnetometer

Attitude estimation can also be achieved by a combination of an accelerometer and a magnetometer. The accelerometer and magnetometer output models can be expressed as follows [25]:

(8) $$\left\{ \begin{array}{l} a^{B}=C_{N}^{B}(q_{I})\left(a_{e}-g^{N}\right)+\mu_{a}\\ m^{B}=C_{N}^{B}(q_{I})(m^{N})+\eta_{m} \end{array}\right.$$

where, $a_{e}$ is the acceleration of the UAV motion, $g^{N}$ is the gravity vector under the N system, and $\mu_{a}$ is the measurement noise of the accelerometer, including white noise and high-frequency vibration noise in the actual application. $m^{N}$ is the geomagnetic field vector in the N system, and $\eta_{m}$ is the measurement noise of magnetometer. It can be seen from the above formula that the accelerometer output is sensitive to the motion acceleration of the UAV. Therefore, this method is generally used when the UAV maintains static or its acceleration changes slowly [26], that is $a_{e}=0$, then the above formula becomes:

(9) $$\left\{ \begin{array}{l} a^{B}=C_{N}^{B}(q_{I})\left(g^{N}\right)+\mu_{a}\\ m^{B}=C_{N}^{B}(q_{I})(m^{N})+\eta_{m} \end{array}\right.$$

There are many ways to achieve attitude estimation by using accelerometers and magnetometers, such as Gradient Descent Algorithm, Newton Iteration Algorithm, etc. In this paper, we choose triad algorithm [27] to estimate the attitude. Its advantage is that its calculation is simple and its output result is quaternion, which is convenient for the subsequent complementary filtering algorithm. The algorithm process is as follows:

Calculate three orthogonal basis vectors of the B system:

(10) $$r_{1}=\frac{a^{B}}{\left\|a^{B}\right\|},r_{2}=\frac{a^{B}\times m^{B}}{\left\|a^{B}\times m^{B}\right\|},r_{3}=r_{1}\times r_{2}$$

Similarly, calculate three orthogonal basis vectors of the N system:

(11) $$s_{1}=\frac{g^{N}}{\left\|g^{N}\right\|},s_{2}=\frac{g^{N}\times m^{N}}{\left\|g^{N}\times m^{N}\right\|},s_{3}=s_{1}\times s_{2}$$

where $s_{i}=C_{B}^{N}\left(q_{I}\right)r_{i}$, define $m_{ref}$ and $m_{mea}$ as follows:

(12) $$M_{ref}=[\begin{array}{ccc} s_{1} & s_{2} & s_{3} \end{array}],M_{mea}=[\begin{array}{ccc} r_{1} & r_{2} & r_{3} \end{array}]$$

then:

(13) $$M_{ref}=C_{B}^{N}(p_{I})M_{mea}$$

Considering $m_{ref}$ and $m_{mea}$ are orthogonal matrices, thus:

(14) $$C_{B}^{N}\left(p_{I}\right)=M_{ref}M_{mea}^{T}$$

where:

(15) $$C_{B}^{N}(p_{I})=\left[\begin{array}{ccc} C_{11} & C_{12} & C_{13}\\ C_{21} & C_{22} & C_{23}\\ C_{31} & C_{32} & C_{33} \end{array}\right]$$

Then, the attitude quaternion can be obtained by the following formula:

(16) $$\{\begin{array}{l} q_{0}=\frac{1}{2}\sqrt{1+C_{11}+C_{22}+C_{33}}\\ \;q_{1}=\frac{1}{4q_{0}(C_{32}+C_{23})}\\ \;q_{2}=\frac{1}{4q_{0}}(C_{13}+C_{31})\\ \;q_{3}=\frac{1}{4q_{0}}(C_{13}+C_{31}) \end{array}$$

### 2.4. Complementary Filtering Algorithm

Gyroscope measurements have high precision in a short time under dynamic conditions, but their static performance is poor due to the influence of drift. In contrast, accelerometers and magnetometers have poor high frequency accuracy, but their measurement errors do not accumulate with time, which means their static performance is good [28]. In view of their complementary characteristics in the frequency domain, the complementary filtering algorithm can be used to fuse their measurements so that improve the accuracy of attitude estimation.

**Figure 1:**
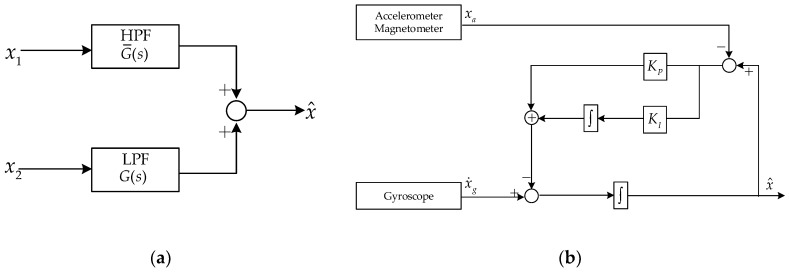
Structures of the linear complementary filtering and nonlinear complementary filtering: (**a**) Structure of CF; (**b**) structure of NCF.

The basic structure of the complementary filtering algorithm is shown in Figure 1a. $x_{1}$ and $x_{2}$ are signals obtained by coupling low frequency noise and high frequency noise to real signals, respectively. Let G(s) be a low-pass filter, then $\bar{G}\left(s\right)=1-G\left(s\right)$ is a high-pass filter, in which s=σ+jω is the plural variable in frequency domain used in Laplace transform. And the output of the complementary filter can be expressed as [29]:

(17) $$\hat{x}=x_{1}*G\left(s\right)+x_{2}*\bar{G}\left(s\right)$$

For attitude estimation, x={γ,θ,ψ} is used to represent the attitude angle of the micro-UAV, ${\dot{x}}_{g}=\left\{\dot{\gamma },\dot{\theta },\dot{\psi }\right\}$ is the attitude estimated by the gyroscope, and $x_{a}=\left\{\gamma_{a},\theta_{a},\psi_{m}\right\}$ is the attitude estimated by the accelerometer/magnet. The linear complementary filter output can be expressed as:

(18) $$\hat{x}=\alpha \left(\int {\dot{x}}_{g}dt\right)+\left(1-\alpha \right)x_{a}$$

The weight coefficient α is between [0, 1], which determines the proportion of the attitude angle estimated by the gyroscope in the final attitude result. Considering that linear complementary filtering is not suitable for nonlinear state estimation and the sensor output drifts with time, a nonlinear complementary filter (NCF) that using PI control to reduce steady-state error and compensate for time drift has been widely used [30]. The structure of NCF is shown in Figure 1b, and its output is expressed as:

(19) $$\hat{x}=\frac{1}{s}\left[{\dot{x}}_{g}+\left(K_{p}+\frac{K_{I}}{s}\right)\left(x_{a}-\hat{x}\right)\right]$$

The nonlinear complementary filtering algorithm can effectively control the high frequency and low frequency noise. Its result is smooth and it need less computation compared with CF. However, there are still several shortcomings in the NCF algorithm: (1) The dynamic adjustment of parameters $K_{p}$ and $K_{I}$ is difficult, and the convergence time and accuracy of NCF are difficult to control. (2) Since Gaussian white noise is distributed in all frequency bands, it is limited for NCF algorithm in the effect of noise suppression:

## 3. Improved Algorithm for NCF

### 3.1. Proposed of First-Order Differential Data Processing Algorithm for Gyroscope

It can be seen from the above analysis that gyroscopes are the main data sources in the complementary filtering attitude estimation, which plays a very important role. The function of accelerometers and magnetometers is to compensate the drift of the gyroscope. Besides, the gyroscope estimates the attitude by integrating the angular velocity, so that noise or abnormal data in gyro data will make the estimation result deviate from the true value with the increase of the integration time. Considering the importance of gyroscopes in attitude estimation, we plan to improve the accuracy of attitude estimation by adding data process of gyroscope.

The core of traditional algorithms for data process of gyroscope is to analyze a lot of gyroscope data in advance, and then design a suitable filter for real-time filtering. It’s obvious that these algorithms have poor portability and flexibility, and the workload is large. Therefore, we consider designing a data processing method for gyroscope with good real-time performance, good flexibility and small amount of calculation which is suitable for micro-UAV.

Because the sampling rate of the gyroscope is much higher than the attitude update frequency, there is time correlation between gyroscope data. Considering that the difference method can recognize rough errors and replace them with reasonable values, the quantile method has strong tolerance to errors. Therefore, in order to improve the reliability of gyroscope data, we design a first-order difference data processing algorithm for gyroscope based on the difference method and quantile method in data processing. The algorithm is shown below.

Assume the data measured by a gyroscope in current time is $\omega_{g}(n)$. Then, the sequence $\left\{\omega_{g}(n-i),i\in \left\{0,1\ldots ,m-1\right\}\right\}$ of length m is obtained by combining $\omega_{g}(n)$ with the previous m−1 measurements. For convenience of illustrating the algorithm, we re-record the sequence as {x(i),i∈{1,2,…,m}}. Calculate the difference sequence by the following formula:

(20) y(i)=x(i+1)−x(i), i∈{1,2,…,m−1}

Get sequence $\left\{y^{\prime }\left(i\right),i\in \left\{1,2,\ldots m-1\right\}\right\}$ by sorting y(i) from largest to smallest. Then, calculate its median M, the upper quartiles $F_{U}$ and lower quartiles $F_{L}$. Then, the quartile dispersion of the sequence is defined as:

(21) $$dF=F_{U}-F_{L}$$

The following equation can be used to judge whether the data $\omega_{g}(n)$ is reliable. In the formula, α is the credibility threshold, the algorithm has better fault tolerance if α selects a large value. In contrast, the algorithm has higher sensitivity if α selects a small value:

(22) |y(m−1)−M|>α∗dF

For a specific α, if the above formula is true, the data $\omega_{g}(n)$ can be considered untrustworthy and it can be corrected by the following formula. On the contrary, if the above formula is not true, then $\omega_{g}(n)$ is considered trustworthy, and the original data can be retained:

(23) $$\omega_{g}\left(n\right)=\omega_{g}\left(n-1\right)+y\left(m-1\right)$$

where, $\omega_{g}(n)$ is the data measured by gyroscope at current time whose subscript g means it is measured in UAV coordinate system. It can be seen that the principle of correction process is re-estimate the current measurement with previous data. Once a new measurement is obtained by gyroscope, it will be processed by this algorithm. Obviously, this algorithm has strong real-time performance.

### 3.2. Design Adaptive Adjustment Strategy for Constants and Proposed of D-NCF

In the above processing algorithm of gyroscope data, two constants α and m are involved. α is the data credibility threshold and m is the length of differential sequence. The choice of their values will greatly affect the gyroscope data processing effect, which in turn affects the effect of the NCF algorithm.

Intuitively, the values of α and m should be related to the dynamic characteristics of the gyroscope signal. Therefore, it is desirable to achieve adaptive adjustment of the value of α and m according to signal changes. In this paper, we choose the following formula to judge the movement of the micro-UAV:

(24) $$d_{g}={\left\|\omega_{g}\left(n\right)\right\|}_{2}^{2}<\varepsilon$$

In the above formula, ε is the threshold depending upon the noise feature of the gyroscope. If the above formula is true for a specific ε, it indicates that the gyro data has a relatively small amplitude change, which means the micro-UAV is currently in a stationary or steady state. In this case, for α we should choose a small value, which can improve the data credibility discrimination, and for m we should choose a large value for the strong correlation between the data; on the contrary, if the above formula is not true, which means the attitude angle of the micro-UAV is changing. In this case, a large value of α should be chosen and m should have a small value.

The value of ε should be determined according to the characteristics of the gyroscope. If the gyroscope has high accuracy and good stability, a small value of ε should be chosen; otherwise, a large value of ε should be chosen. In the Section 4.2.1, we give an introduction to the method of selecting the value of ε.

**Figure 2:**
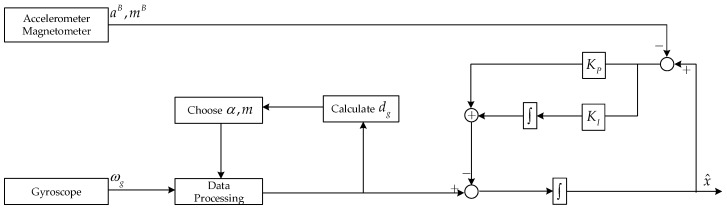
The structure of D-NCF algorithm.

By combining the first-order differential data processing algorithm mentioned in Section 3.1 with the basic NCF algorithm, a new improved algorithm is proposed in this paper, which is named D-NCF. Its structure is shown below (Figure 2).

According to the process of first-order differential data processing algorithm. We can know the pseudo flow of D-NCF is as follows:

1. Note the current measurements of the gyroscope, accelerometer, and magnetometer which are $\omega_{g}^{}(n)$, $a^{B}\left(n\right)$, and $m^{B}\left(n\right)$, respectively.
2. Depending on the α and m values calculated before, the gyroscope measurements are processed by the first-order differential data processing algorithm.
3. The attitude angle ${\dot{x}}_{g}$ is obtained by solving the dynamic differential equation according to the gyroscope measurements, and the attitude angle $x_{a}$ is obtained by using the triad algorithm according to the accelerometer and magnetometer measurements. Then, using the NCF algorithm to achieve the fusion of ${\dot{x}}_{g}$ and $x_{a}$, we get the comprehensive attitude angle {γ,θ,ψ}.
4. Calculate $d_{g}$ according to $\omega_{g}^{}(n)$, and then update α,m to prepare for processing the next gyroscope measurement.

In general, the main contributions of this paper in improving of NCF can be divided into two parts: (1) We design a first-order difference data processing algorithm for gyroscopes based on the difference method and quantile method in data processing. (2) The data processing algorithm is used in attitude estimation by combining it with NCF, and the dynamic adjustment strategy for constants is designed.

## 4. Experimental Verification of the D-NCF Algorithm

### 4.1. Experimental Setup

**Figure 3:**
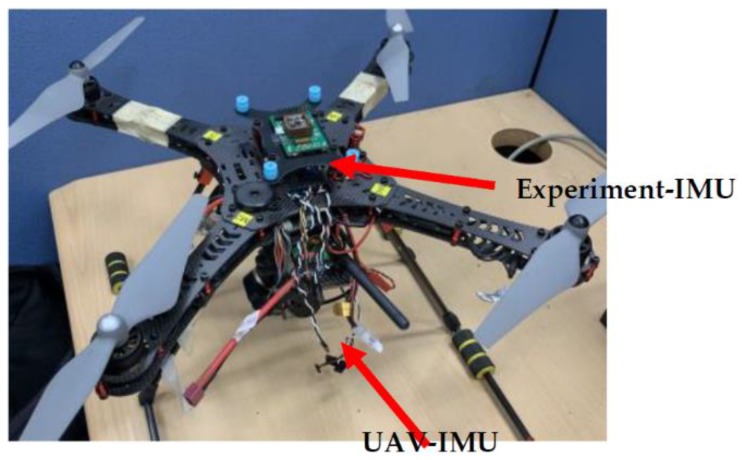
Experimental setup for the measurements collecting from UAV-IMU and Experiment-IMU.

In order to verify the performance of the D-NCF algorithm proposed in this paper, a low-cost, low-precision IMU (experiment-IMU) is mounted on a quad-rotor UAV for experimental data acquisition (see Figure 3). The quad-rotor UAV itself carries a high-precision IMU (UAV-IMU) for flight control. It uses the EKF algorithm for attitude estimation, whose estimation accuracy is high. Therefore, the real-time attitude angle estimated by UAV-IMU is recorded in the experiment, and it is used as a standard to compare the performance of different algorithm. Before the flight experiment, the two IMUs were synchronized in advance, and their data sampling rate was adjusted to 25 Hz. 

The performance comparisons of accelerometers, gyroscopes, and magnetometers contained in the two IMUs are shown in Table 1. It’s obvious that UAV-IMU is significantly superior to the experiment-IMU in performance.

### 4.2. Experiment Results and Discussion

The experiment-IMU and UAV-IMU both have sensor calibration and reset functions. After the quad-rotor UAV is stable in the air, the two IMUs are manually reset through the Bluetooth interface present on them, and this moment is recorded as 0. After 180 s, we let the micro-UAV turn a small angle, and then keep it stable. After about 800 s, the UAV is instructed to land, and we read the data from two IMUs. A total of 1272 data were obtained. Then, we perform algorithm processing and verification in Matlab.

#### 4.2.1. First-Order Differential Data Processing Algorithm for Gyroscope

The first-order differential data processing algorithm is performed on the x-y-z triaxial measurements of the gyroscope in experimental-IMU. The first step is to determine the value of ε. When the UAV is stable in the air, we collect 200 datapoints of gyroscopes in x-y-z triaxial coordinates for observation. We can find that the value of all the data is less than 0.1 though there is small drift with time. Considering $0.1^{2}\times 3=0.03$, add 0.01 for redundancy, then $\sqrt{0.04}=0.2$. Thus, the value of ε is selected as 0.2 in this experiment.

The value of $d_{g}$ is then updated in real time and the value of α,m is dynamically adjusted according to the magnitude of $d_{g}$.Through many attempts, the most suitable adjustment strategy for this experiment was designed (see Table 2).

**Figure 4:**
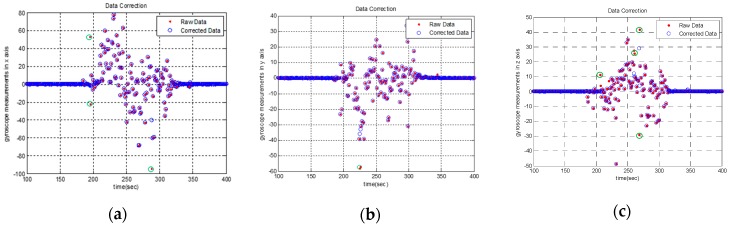
Show of gyroscope raw data and the corrected data by applying first-order differential data processing algorithm: (**a**) gyroscope measurements in x axis; (**b**) gyroscope measurements in y axis; (**c**) gyroscope measurements in z axis.

Results are shown in Figure 4. In the figure, the point marked by the green circle is the point that the algorithm believes that its credibility is insufficient. These points have been greatly revised in magnitude. 

Because the sampling rate of the gyroscope is much higher than the attitude update frequency, there is time correlation between gyroscope normal data. On the contrary, coarse and abnormal data are far from normal data and are not time-dependent with previous and subsequent data. Therefore, points farther away from the axis are likely to be error data. It can be saw from Figure 4. that most of points which revised by the first-order differential data processing algorithm are highly deviated, and most likely are unreliable data, which indicates that the reliability of the modified algorithm is relatively high.

#### 4.2.2. Attitude Estimation Accuracy of D-NCF Algorithm

**Figure 5:**
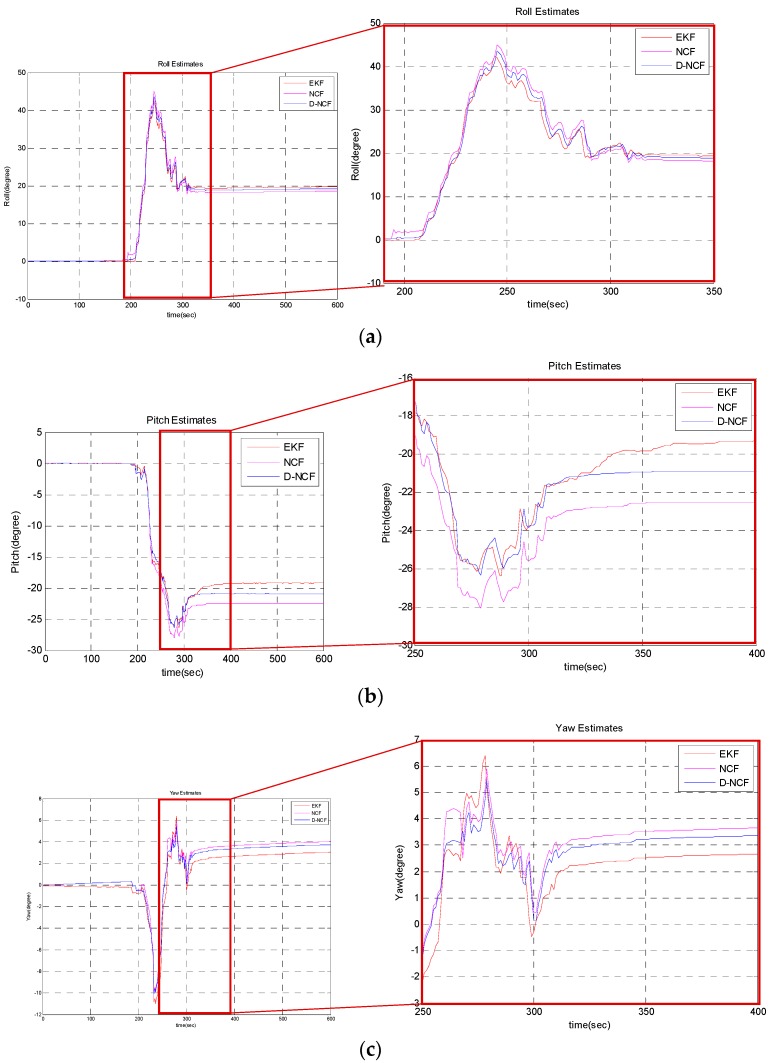
Comparison of attitude estimation accuracy between EKF, NCF and D-NCF algorithm: (**a**) estimated result of roll angle by using EKF, NCF and D-NCF algorithm respectively; (**b**) estimated result of pitch angle by using EKF, NCF and D-NCF algorithm respectively; (**c**) estimated result of yaw angle by using EKF, NCF and D-NCF algorithm respectively.

Separately use NCF algorithm and D-NCF algorithm to estimate the attitude on the measurements collected by the experimental-IMU. In both algorithms, the same parameters (T = 0.0007; Kp = 0.04; Ki = 0.03) are selected. In this experiment, attitude estimate result of UAV-IMU is used as a standard to compare the performance of NCF and D-NCF. In order to better illustrate algorithm effect, some of the result are intercepted and enlarged. The result is shown in Figure 5. It can be saw that the result of the D-NCF algorithm is closer to the result of the EKF algorithm, which means that attitude estimation accuracy of D-NCF is higher than that of the basic NCF algorithm. However, it only qualitatively describes the performance of D-NCF algorithm, and there is a lack of quantitative description here.

The Root Mean Square Error (RMSE) can be used to measure how far the data deviates from the true value. In order to better compare the accuracy between NCF and D-NCF algorithm, we choose the RMSE as the measurement method of attitude estimation error. The formula for calculating the RMSE is defined as follows:

(25) $$RMSE={\left\|\overset{\wedge }{x}-x\right\|}_{2}^{2}/\sqrt{N}$$

where, x represents the attitude estimated by UAV-IMU according to the EKF algorithm, and $\hat{x}$ represents the attitude estimated by experiment-IMU according to NCF algorithm or D-NCF algorithm. N is the amount of data. After calculation, the estimation error results of the three attitude angles are shown in Table 3. It can be clearly seen that the estimation error of the D-NCF algorithm is smaller than the NCF algorithm. The attitude estimation error of roll angle decreases from 1.1653 to 0.5093, pitch angle decreases from 2.9638 to 1.5542, and yaw angle decreases from 0.9398 to 0.6827.

Combining Figure 5 and Table 3 for analysis, we can find that the attitude estimation accuracy of D-NCF algorithm is higher than NCF algorithm. However, the amount of sampling data is limited which means the overall characteristics of the D-NCF algorithm are not fully described. In order to make consistent the conclusions, consider carrying out hypothesis contrast test for statistical demonstrated.

Take two groups of samples, X1 and X2, whose sample size is n=160. X1 and X2 represent the differences between NCF and D-NCF attitude angles respect to EKF reference separately. They are defined as follows:

X1 = attitude angle (NCF)-attitude angle (EKF), X2 = attitude angle (D-NCF)-attitude angle (EKF)M(26)

After comparison, Z-Test was selected for statistical demonstration. Define $\mu_{1}$ is the mean of sample X1, and $\mu_{2}$ is the mean of sample X2. Then, we make two hypotheses:

(27) $$\begin{array}{c} H_{0}:\mu_{1}\mathrm{and}\;\mu_{2}\;\mathrm{are}\;\mathrm{not}\;\mathrm{significantly}\;\mathrm{different}\\ H_{1}:\mu_{1}\mathrm{and}\;\mu_{2}\;\mathrm{are}\;\mathrm{significantly}\;\mathrm{different} \end{array}$$

The statistic value of the Z-Test is:

(28) $$Z=\frac{\mu_{1}-\mu_{2}}{\sqrt{\frac{s_{1}^{2}+s_{2}^{2}}{n}}}$$

where, $s_{1}$ and $s_{2}$ are the standard deviations of X1 and X2. After calculation, the results of Z are shown in Table 4. It can be seen that the absolute value of Z in the three attitude angles are all greater than 2.58, indicating that the hypothesis $H_{1}$ is true which means $\mu_{1}$ and $\mu_{2}$ are significantly different. Combined with the previous conclusions, it is further explained that the overall effect of D-NCF algorithm in attitude estimation is better than NCF algorithm, and the effect is improved significantly.

Since flight control requires high real-time performance of the attitude estimation algorithm, the real-time performance of D-NCF algorithm is tested here. By using the data collected by the experimental-IMU, we used the NCF and D-NCF algorithms, respectively, to successively perform 1272 attitude estimation. Their attitude estimation time is shown in Table 5. According to the three experimental results, we can find that D-NCF algorithm has a good real-time performance, and only adds about 0.01 s processing time. It can thus meet the requirements of flight control.

#### 4.2.3. Robustness of D-NCF Algorithm

**Figure 6:**
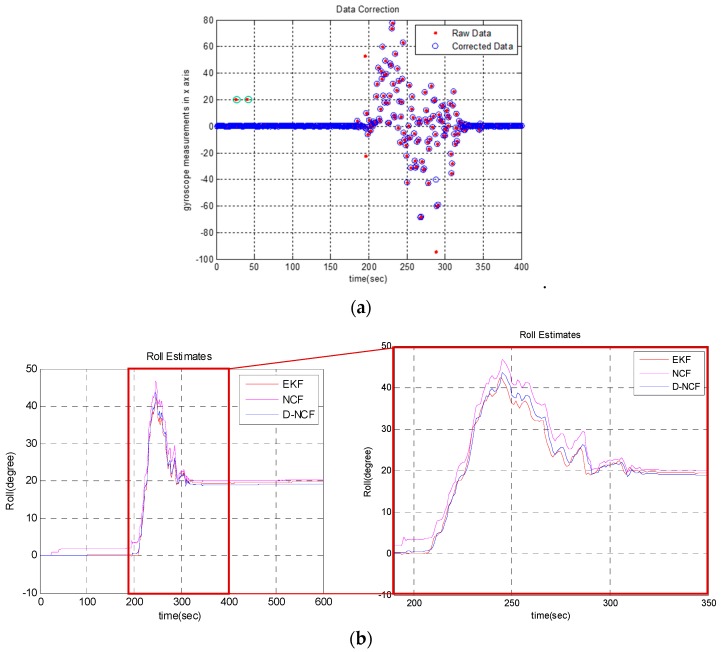
Examine result of robustness of D-NCF algorithm: (**a**) Processing result of the error data by using first-order differential data processing algorithm; (**b**) estimated result of roll angle after adding the error data.

In order to examine the robustness of the D-NCF algorithm, in this experiment, we artificially add two erroneous datapoints into the gyroscope x-axis measurement at t = 25 and t = 40. Then we separately use the NCF algorithm and D-NCF algorithm to estimate the roll angle again. Figure 6 shows the results. It can be seen that with the general NCF algorithm it is easy to introduce erroneous data into the attitude estimation due to the lack of discrimination on the data credibility, which ultimately leads to inaccurate attitude estimation. In comparison, the D-NCF algorithm can realize the recognition and correction of erroneous data, which can guarantee the correctness of the attitude estimation.

### 4.3. Discussion of Experiment

The above experimental results can demonstrate the contribution of proposed D-NCF algorithm. In Section 4.2.1, the first-order differential data processing algorithm is performed on the x-y-z triaxial measurements of the gyroscope in experimental-IMU. From Figure 4, we can intuitively find that the algorithm is reliable in processing gyroscope data. In Section 4.2.2, we separately use the NCF algorithm and D-NCF algorithm to estimate the attitude on the measurements collected by the experimental-IMU. Figure 5 shows the overall effect of D-NCF algorithm for attitude estimation. Then RMSE is chosen to indicate the accuracy of the algorithm in attitude estimation. Besides, in order to make consistent the conclusions, a hypothesis contrast test is carried out for statistical demonstration. It can be seen from the above results that the attitude estimation accuracy of D-NCF algorithm is higher than that of the NCF algorithm. At the same time, the attitude estimation time of D-NCF algorithm and NCF algorithm is shown in Table 5. It shows that D-NCF algorithm has good real-time performance. In Section 4.2.3, we test the robust performance of the D-NCF algorithm. Results illustrate that the D-NCF algorithm can realize the recognition and correction of erroneous data, which can guarantee the correctness of the attitude estimation. In general, the experiment of three sections proves that D-NCF algorithm has better performance than the traditional NCF algorithm in attitude solution of micro-UAVs.

However, in the D-NCF algorithm proposed in this paper, the choice of α and m will greatly affect the effect of the algorithm. Although the paper has designed the adaptive adjustment strategy for them, in this experiment, α and m are selected by multiple attempts and only two cases ($d_{g}>\varepsilon$ and $d_{g}\leq \varepsilon$) are considered. This may influence the performance of the D-NCF algorithm and it can’t achieve its best results. In subsequent studies, it may be considered to subdivide the two cases and design the values of α and m by a fuzzy algorithm [31].

## 5. Conclusions

In micro-UAV attitude estimation applications, a good algorithm should be balanced between the amount of calculation and precision. Compared with other attitude estimation algorithms, NCF algorithms has the smallest amount of calculation, which is suitable for attitude estimation of micro-UAVs whose computing resources are limited. However, the accuracy of the NCF algorithm is low, which may affect the attitude control accuracy. Therefore, under the premise that the amount of calculation does not increase significantly, improving the accuracy of NCF algorithms is an urgent problem need to be solved.

Considering that the gyroscope data is the core of the complementary filtering algorithm, and the gyroscope data needs to be integrated in the attitude estimation, which means the reliability and accuracy of the gyroscope data will greatly affect the complementary filtering performance. Therefore, in view of the difference method and quantile method in data processing, this paper designs a first-order differential data processing algorithm for gyroscope data. It is suitable for gyroscopes with less computation and better real-time performance. We propose the D-NCF algorithm by combining it with the basic NCF algorithm. In general, the main contribution of the paper in improving of NCF can be divided into two parts: (1) Design a first-order difference data processing algorithm for gyroscope based on the difference method and quantile method in data processing. (2) The data processing algorithm is used in attitude estimation by combining it with NCF, and the dynamic adjustment strategy for constants is designed.

The experimental results show that the D-NCF algorithm can effectively correct the gyroscope data and improve the accuracy of the complementary filtering. The attitude estimation error of the roll angle decreases from 1.1653 to 0.5093, that of the pitch angle decreases from 2.9638 to 1.5542, and that of the yaw angle decreases from 0.9398 to 0.6827 at the cost of increasing the calculation time by approximately 0.01 s compared to the NCF algorithm. It can meet the requirements of flight control. Besides, the D-NCF algorithm can realize the recognition and correction of erroneous data, which can guarantee the correctness of the attitude estimation. In general, the proposed algorithm is valuable in micro-UAV attitude estimation applications.

## Figures and Tables

**Table 1:** Performance comparison of accelerometers, gyroscopes, and magnetometers contained in the experimental IMU and UAV-IMU.

		Experiment-IMU	UAV-IMU
accelerometers	Dynamic Range	±4 g	±16 g
	Digital Resolution	0.244 mg/LSB	0.122 mg/LSB
	Noise Density	90 $\mu g/\sqrt{\mathrm{Hz}}$	90 $\mu g/\sqrt{\mathrm{Hz}}$
gyroscopes	Dynamic Range	±500 deg/s	±245 deg/s
	Digital Resolution	8.75 mdps/LSB	4.375 mdps/LSB
	Noise Density	9 $\mathrm{mdps}/\sqrt{\mathrm{Hz}}$	7 $\mathrm{mdps}/\sqrt{\mathrm{Hz}}$
magnetometers	Dynamic Range	±12 gauss	±8 gauss
	Digital Resolution	3421 LSB/gauss	6842 LSB/gauss
	Noise Density	2 mGa	2 mGa

**Table 2:** The choose of α and m according to the value of $d_{g}$.

	α	m
$d_{g}={\left\|\omega_{g}\left(n\right)\right\|}_{2}^{2}<0.2$	6	13
$d_{g}={\left\|\omega_{g}\left(n\right)\right\|}_{2}^{2}>0.2$	8	9

**Table 3:** Attitude estimation error of NCF and D-NCF algorithm in roll, pitch and yaw.

	NCF	D-NCF
Roll	1.1653	0.5093
Pitch	2.9638	1.5542
Yaw	0.9398	0.6827

**Table 4:** The value of Z in roll, pitch and yaw.

	Roll	Pitch	Yaw
Z	3.020	−20.5024	4.6646

**Table 5:** The attitude estimation time of NCF and D-NCF.

	NCF	D-NCF
1	0.0410 s	0.0517 s
2	0.0440 s	0.0553 s
3	0.0504 s	0.0617 s
